# Set1 cooperates with CENP-B in genome organization and transcriptome regulation

**DOI:** 10.1186/1756-8935-6-S1-P12

**Published:** 2013-03-18

**Authors:** David R Lorenz, Irina V Mikheyeva, Lauren Meyer, Peter Johansen, Anastasia Berg, Shiv IS Grewal, Hugh P Cam

**Affiliations:** 1 Boston College, Biology Department Chestnut Hill, MA 02467, USA; 2 Laboratory of Biochemistry and Molecular Biology National Cancer Institute, National Institutes of Health, Bethesda, MD 20892, USA

## 

Chromatin modifiers impose regulatory controls over diverse chromosomal processes including transcription, nuclear organization, and genome stability. Here we reveal an unexpected role for the histone methyltransferase Set1 (KMT2) as a general transcriptional repressor of the fission yeast *Schizosaccharomyces pombe* genome. Set1 localizes to repetitive elements and represses both forward and reverse transcripts associated with centromeric and subtelomeric heterochromatin and *Tf2* long terminal repeats (LTRs) retrotransposons distinct from its H3K4 methylation (H3K4me) activity. Set1 cooperates with Abp1, the *S. pombe* homolog of the mammalian centromere binding protein B (CENP-B), to mediate its repression of Tf2s. Intriguingly, Set1 helps organize dispersed *Tf2s* into distinct nuclear foci termed *Tf* bodies, the integrity of which requires class I/II histone deacetylases (HDACs) and Sirtuin. Our study uncovers dual roles for Set1 in the maintenance of euchromatin and heterochromatin, and its cooperation with a functional transposase-containing transcription factor to mediate repression and genome organization of dispersed retrotransposons.

